# Immortalized mesenchymal stromal cells overexpressing alpha‐1 antitrypsin protect acinar cells from apoptotic and ferroptotic cell death

**DOI:** 10.1111/jcmm.70093

**Published:** 2024-10-28

**Authors:** Sara Shoeibi, Erica Green, Hua Wei, Wenyu Gou, Charlie Strange, Hongjun Wang

**Affiliations:** ^1^ Department of Surgery Medical University of South Carolina Charleston South Carolina USA; ^2^ Department of Medicine Medical University of South Carolina Charleston South Carolina USA; ^3^ Ralph H. Johnson Veterans Affairs Medical Center Charleston South Carolina USA

**Keywords:** acinar cell death, chronic pancreatitis, ferroptosis, immortalized human MSCs

## Abstract

Chronic pancreatitis (CP) is a progressive inflammatory disorder that impairs endocrine and exocrine function. Our previous work showed that mesenchymal stem/stromal cells (MSCs) and MSCs overexpressing alpha‐1 antitrypsin (AAT‐MSCs) could be therapeutic tools for CP. However, primary MSCs are predisposed to undergo senescence during culture expansion, which limits their therapeutic applications. We generated and characterized immortalized human MSCs (iMSCs) and AAT‐MSCs (iAAT‐MSCs) and tested their protective effect on 2,4,6‐Trinitrobenzenesulfonic acid (TNBS)‐induced acinar cell death in an in vitro cell culture system. Primary MSCs were immortalized by transduction with simian virus 40 large T antigen (SV40LT), and the resulting iMSC and iAAT‐MSC lines were analysed for proliferation, senescence, phenotype and multi‐differentiation potential. Subsequently, apoptosis and ferroptosis pathways were investigated by assessing changes before and after TNBS treatment. Coculture of iMSCs and iAAT‐MSCs with acinar cell lines inhibited early cell death induced by TNBS, reduced ER stress and reversed TNBS‐induced protein reduction at tight junctions. Additionally, iMSCs and iAAT‐MSCs exerted such protection by regulating mitochondrial respiration, ATP content and ROS production in TNBS‐induced acinar cells. Furthermore, iMSCs and iAAT‐MSCs ameliorated TNBS‐induced ferroptosis by modulating iron generation and ROS production and regulating the ferritin heavy chain 1 (FTH1)/protein disulfide isomerase (PDI)/glutathione peroxide 4 (GPX4) signalling pathways in acinar cells. These findings identify ferroptosis as an unrecognized mechanism that leads to TNBS‐induced cell death and offer mechanistic insights relevant to using stem cell therapy to treat acinar cell death associated with CP.

## 1 INTRODUCTION

Chronic pancreatitis (CP) involves inflammatory cell recruitment, activation of pancreatic stellate cells, subsequent acinar cell apoptosis and necrosis and fibrosis.^1^, ^2^ Because pancreatic acinar cells constitute the largest population of parenchymal cells in the pancreas, the accumulation of excess amino acids in these cells in response to hormonal stimulation may cause pancreatic toxicity and contribute to the onset and progression of CP by mechanisms that are not yet fully understood.^3^, ^4^ Alterations in cellular pathways in the acinar cells, a major cell type of exocrine pancreas, offer insights into the molecular events that occur during the early stages of CP.^4^, ^5^ Identifying the underlying cellular mechanisms involved in acinar cell death in CP is crucial for understanding its pathophysiology and developing novel therapeutic options. Apoptosis plays a crucial role in CP, destroying acinar cells and other cells in the pancreas, leading to the disruption of exocrine function. Oxidative stress is also implicated in the pathogenesis of CP, as increased generation of superoxide anions (O^2−^) and reactive oxygen species (ROS) are chronically activated in CP.^6^, ^7^ The main effectors of apoptosis, such as caspases and the B cell lymphoma 2 (Bcl‐2) family, may regulate the progression of CP by linking the outer and inner pathways that promote apoptosis.^7^, ^8^ While many studies have examined the role of apoptosis in various pancreatitis models, the specific cell death pathways, and their relationship to acinar cell loss in CP remain unclear.^9^, ^10^, ^11^, ^12^

Ferroptosis is a regulated form of cell death associated with lipid peroxidation and disrupted redox homeostasis.^13^, ^14^, ^15^ The process of ferroptosis is triggered by intracellular iron overload and the inactivation of glutathione peroxide 4 (GPX4).^13^, ^16^ Although ferroptosis has been investigated in the development of various diseases, such as ischemia–reperfusion injury,^13^ ulcerative colitis^14^ and colorectal cancer,^15^ its role in the development of chronic pancreatitis has not been reported. A recent study demonstrated that the downregulation of GPX4 by the transcription factor AP‐1 is critical in the aggravation of acinar cell ferroptosis during the progression of acute pancreatitis.^17^ Whether ferroptosis is involved in acinar cell death in CP has yet to be studied. Therefore, we hypothesize that ferroptosis plays a critical role in TNBS‐induced acinar cell death, and inhibiting ferroptosis may be a potential therapeutic option for treating CP and other diseases associated with ferroptosis.

Mesenchymal stromal cells (MSCs) have therapeutic potential due to their immunoregulatory and anti‐inflammatory effects.^3^, ^18^, ^19^, ^20^ Overexpressing alpha‐1 antitrypsin (AAT) in MSCs shows promise in anti‐inflammatory properties.^19^, ^21^, ^22^, ^23^ However, like other adult stem cells, MSCs undergo telomere shortening with each cell division. Various types of cellular stress can lead to chromosomal instability, DNA damage accumulation and the acquisition of a senescent phenotype.^24^ Several studies have shown that culture‐expanded MSCs have decreased proliferation, lower expression of specific cell surface markers, limited differentiation potential, and are prone to senescence in vitro, thus compromising their therapeutic usefulness.^24^, ^25^, ^26^ To overcome in vitro senescence of aged MSCs, lines of immortalized MSCs (iMSCs) have been created by transducing immortalizing genes such as simian virus 40 large T antigen (SV40LT) and human papillomavirus E6/E7 genes, which have attracted significant research interest.^24^, ^27^ It is believed that genetic alterations that promote cell cycle progression and suppress stress‐induced senescence are required for MSC immortalization.^25^, ^27^ Immortalizing MSCs with immortalizing genes is a promising approach to overcoming senescence.^24^, ^27^, ^28^

This study generated immortalized MSC lines (iMSCs and iAAT‐MSCs) and investigated their effects on apoptosis and ferroptosis induced by TNBS in acinar cell lines. iMSCs and iAAT‐MSCs inhibited TNBS‐induced ferroptosis by suppressing endoplasmic reticulum (ER) stress and ROS production. These immortalized MSCs hold promise for CP cell therapy.

## 2 MATERIALS AND METHODS

### 2.1 Cell culture

Rat pancreatic AR42J acinar cells (pancreatoma, ATCC CRL‐1492) were cultured in F‐12 K medium (ATCC, USA) supplemented with 20% fetal bovine serum (FBS; Thermo Fisher Scientific, MA, USA) and 1% penicillin/streptomycin (Gibco, MT, USA) at 37°C with 5% CO_2_ until they reached over 80% confluency. Cells were detached using 0.25% trypsin solution containing 2.2 mM EDTA (Gibco, MT, USA). Human bone marrow‐derived MSCs were derived from bone marrow specimen purchased from Stemexpress (Folsom, CA, USA). The donor was a 25‐year‐old healthy African American male. MSCs were separated from bone marrow as described previously,^29^ Cells were cultured in low glucose DMEM (Gibco, MT, USA) supplemented with 10% FBS and 1% penicillin/streptomycin at an initial density of 5000 cells/cm^2^.

We generated human AAT‐engineered MSCs (hAAT‐MSCs) by transducing hMSCs with the pHAGE‐CMV‐a1aT‐UBC‐GFP‐W lentiviral vectors as previously described.^22^ MSCs transduced with the control virus, pHAGE‐UBC‐GFP‐W, were used as control. Following viral infection, more than 80% of MSCs were GFP^+^, indicating successful transfection. Positive cells were then sorted by flow cytometry based on GFP expression and used to generate immortalized cells.

### 2.2 Immortalization of primary MSCs and AAT‐MSCs

We generated immortalized cell lines of MSCs and AAT‐MSCs using SV40 T antigen (ALSTEM, CA, USA) at passages 4–5. The target cells were transduced with 20 μL/well of SV40LT viral supernatant in the presence of 4 μL TransPlus reagent and selected using puromycin (Gibco, MT, USA). Two weeks after selection, clones were chosen for expansion and screening. MSCs and AAT‐MSCs at P8 or P9 and iMSCs and iAAT‐MSCs at P18‐P20 were used in this study.

### 2.3 Coculture of iMSC and iAAT‐MSC with AR42J cell lines

AR42J cells, a proliferative pancreatic acinar cell line, were seeded in the bottom well of the Transwell 6‐well plates (Corning, NY, USA) at a density of 0.3 × 10^6^ cells/cm^2^. iMSCs or iAAT‐MSCs at a density of 0.6 × 10^6^ cells/cm^2^ were added on the insert of Transwells in a complete medium for 24 h. The medium was then changed to DMEM supplemented with 5% FBS and TNBS at 0.10%, or 0.15% for controls (TNBS only) and two treatment groups (TNBS + iMSCs or iAAT‐MSCs). Cells cultured alone without TNBS were used as healthy cell controls. After 24 h of coculture, AR42J cells were collected for further analysis.

### 2.4 Microscopic analysis

#### 2.4.1 Senescence‐associated β‐galactosidase (β‐gal) activity

Senescence assay was performed on iMSCs and iAAT‐MSCs (passage 18, P18), primary MSCs, and AAT‐MSCs (P9) using the Senescence β‐Galactosidase Staining kit (Cell Signaling Technology, MA, USA). Cells were incubated overnight in β‐gal staining solution (final concentration 1 mg/mL, pH 6.0). The percentage of senescent cells was determined by counting ß‐Gal‐positive and total cells in five randomly selected microscope fields.

#### 2.4.2 Colony‐forming unit‐fibroblast assay (CFU‐F)

In the colony formation assay, MSCs were plated in triplicate at densities of 50, 100 and 200 cells per 35 mm well. After 2 weeks of incubation with regular medium replacements, cells were washed, fixed with ice‐cold 100% methanol (Thermo Fisher Scientific, MA, USA), and stained with 0.5% crystal violet (Sigma‐Aldrich, MO, USA). CFU‐F colonies larger than 3 mm in diameter were counted under a light microscope.

#### 2.4.3 Preservation of multipotency in iMSCs and iAAT‐MSCs

MSCs, AAT‐MSCs (P9), iMSCs and iAAT‐MSCs (P20) were subjected to chondrogenic, osteogenic and adipogenic differentiation assays using specific kits from Gibco (MT, USA). Differentiation was induced by culturing the cells in specific differentiation media for 14 days (adipogenic) or 22 days (osteogenic and chondrogenic), with regular medium replacements. Subsequently, staining methods were employed to assess differentiation outcomes: 0.5% Oil Red‐O solution (Sigma‐Aldrich, MO, USA) for adipogenesis, 2% Alizarin red S (pH 4.2, Sciencell, CA, USA) for osteogenesis and 1% Alcian blue (Lifeline Cell Technology, CA, USA) for chondrogenesis following protocols recommended by the manufacturers.

#### 2.4.4 Detection of intracellular iron

Prussian blue staining was performed to evaluate intracellular iron content. AR42J cells were cultured with or without iMSCs/iAAT‐MSCs for 24 h. Subsequently, the cells were treated with 0.15% TNBS for an additional 24 h. After washing with distilled water, the cells were stained with iron solution according to the manufacturer's instructions (Iron Stain Kit; Abcam, MA, USA). A nuclear fast red solution was used for counterstaining. Using a light microscope, the percentage of iron‐positive cells was determined by examining five randomly selected fields of view per sample.

#### 2.4.5 Immunocytochemistry

To assess the expression of SV40LT and GPX4, cells were fixed with 4% paraformaldehyde, permeabilized with 0.3% Triton X‐100 and blocked with 1% bovine serum albumin (Thermo Fisher Scientific, MA, USA). Immunostaining was performed using specific primary and secondary antibodies listed in Table 1. Finally, samples were mounted, and fluorescence micrographs were captured using a Confocal Leica TCS SP5 X microscope (Leica‐microsystem, Wetzlar, Germany).

**TABLE 1:** Unconjugated and conjugated antibodies (with fluorescein isothiocyanate (FITC) or phycoerythrin (PE)) employed for the current study.

Antibody	Specificity	Technique	Source	Dilution
Rabbit Anti‐SV40 Large T Antigen	Transgenic levels of total SV40 Large T Antigen protein	IC, WB	Cell Signaling Technology	IC‐1:100, WB‐1:1000
PE Mouse Anti‐human CD29	Human integrin β1 (ITGB1)	FC	BioLegend	1:100
PE Mouse Anti‐Human CD44	Homing cellular adhesion molecule (HCAM)	FC	BD Pharmingen	1:100
PE Mouse Anti‐Human CD90	Thymocyte differentiation antigen 1 (Thy‐1)	FC	BD Pharmingen	1:100
PE Mouse Anti‐Human CD45	Leukocyte common antigen (LCA)	FC	BD Pharmingen	1:100
Rabbit Anti‐BAX	N‐terminus (N‐20) of Bax	WB	Santa Cruz Biotechnology	1:1000
Rabbit Anti‐Cleaved Caspase‐3	Asp175 of Caspase‐3	WB	Cell Signaling Technology	1:1000
Rabbit Anti‐Cleaved PARP	Asp214 of PARP	WB	Cell Signaling Technology	1:1000
Mouse Anti‐p53	Ser20 of p53	WB	Cell Signaling Technology	1:1000
Rabbit Anti‐p38	p38α, −β or ‐γ MAPK protein	WB	Cell Signaling Technology	1:1000
Rabbit Anti‐JNK/SAPK	JNK1, JNK2 or JNK3 protein	WB	Cell Signaling Technology	1:1000
Rabbit Anti‐Occludin Polyclonal	‐	WB	Thermo Fisher Scientific	3 μg/mL
Rabbit Anti‐ZO‐1 Polyclonal	‐	WB	Proteintech	1:5000
Rabbit Anti‐FTH1	Amino‐terminal region of FTH1	WB	Cell Signaling Technology	1:1000
Rabbit Anti‐PDI	C81H6 of PDI	WB	Cell Signaling Technology	1:1000
Rabbit Anti‐GPX4 Polyclonal	‐	IC	Thermo Fisher Scientific	1:50
Rabbit Anti‐β‐Actin	Cytoplasmic Actin isoforms, β‐Actin and cytoplasmic γ‐Actin	WB	Cell Signaling Technology	1:1000
Goat Anti‐Rabbit IgG, HRP‐linked Antibody	Rabbit polyclonal and monoclonal antibodies	WB	Cell Signaling Technology	1:1000
Horse Anti‐Mouse IgG, HRP‐linked Antibody	Mouse polyclonal and monoclonal antibodies	WB	Cell Signaling Technology	1:1000
Goat anti‐Rabbit IgG Cross‐Adsorbed Secondary Antibody, Alexa Fluor™ 488	Rabbit polyclonal and monoclonal antibodies	IC	Thermo Fisher Scientific	1:500
Goat anti‐Rabbit IgG Highly Cross‐Adsorbed Secondary Antibody, Alexa Fluor™ 568	Rabbit polyclonal and monoclonal antibodies	IC	Thermo Fisher Scientific	1:500

#### 2.4.6 MSC surface marker expression in iMSCs and iAAT‐MSCs by flow cytometry

For flow cytometric analysis, iMSCs and iAAT‐MSCs were detached, washed and incubated with PE‐labelled antibodies against CD29, CD90, CD44 and CD45 (Table 1) in a FACS buffer (Thermo Fisher Scientific, MA, USA). After washing, cells were analysed using a Cytoflex flow cytometer (Beckman Coulter, IN, USA), and FlowJo software (BD Biosciences) was used for data analysis. The results are presented as a percentage of positive cells compared to the non‐stained control cells.

#### 2.4.7 Cell apoptosis assay by flow cytometry

Cell apoptosis was assessed using an Annexin V FITC Apoptosis Detection kit (BioLegend, CA, USA). Cells were harvested, washed and mixed with FITC‐conjugated APC Annexin V (2.5 μL) and propidium iodide solution (5 μL) and incubated for 20 min in the dark at room temperature. The stained cells were then analysed with flow cytometry.

#### 2.4.8 Detection of ROS production

To detect ROS production, the intensity of green fluorescence emitted by converting rhodamine 123 upon reaction with ROS was measured as previously described.^30^ In brief, after treatment with TNBS, AR42J cells were incubated with 5 μM dihydrorhodamine (DHR)‐123 (Sigma‐Aldrich, MO, USA). Cells were collected, and ROS production was quantified using flow cytometry. Fluorescence was measured at 485/528 nm on a Bio‐Tek (VT, USA) spectrophotometer after 20 min of incubation and every 20 min after the first measurement to identify the maximal production of reactive nitrogen oxide species (RNOS)/ROS.

#### 2.4.9 Measurements of mitochondrial respiratory activity

After treatment with TNBS, AR42J cells were transferred to an XF96 culture plate at a density of 8 × 10^3^ cells per well. Oxygen consumption rate (OCR) and extracellular acidification rate (ECAR) were measured using the Seahorse XF analyzer (Agilent Technologies, CA, USA). The XFp Cell Mito Stress A test kit was used for OCR measurements, involving different cycles of injections of oligomycin, FCCP and rotenone/antimycin A. ECAR was measured using a program that included glucose, oligomycin and 2‐deoxy‐D‐glucose injections.^28^

#### 2.4.10 Glutathione peroxidase activity (GPX) assay

GPx activity was analysed using a colorimetric assay kit (Abcam, MA, USA) following the manufacturer's protocol. Briefly, AR42J cells were collected after treatment, and incubated with glutathione reductase (GR) and reduced glutathione (GSH). GPx activity was assessed by adding cumene hydroperoxide and measuring the absorbance at 340 nm.

#### 2.4.11 Lipid peroxidation assay

The relative concentration of malondialdehyde (MDA) was determined in AR42J cell lysates using the Lipid Peroxidation Assay Kit (Abcam, MA, USA). The assay involved reacting MDA with thiobarbituric acid, which was quantified at 352 nm using a microplate reader.

### 2.5 Molecular analysis

Approximately 1.2 × 10^6^ cells were used to extract total cellular RNA with an RNA extraction kit (Qiagen, MD, USA). A 1 μg aliquot of the extracted RNA was used for a reverse transcription‐polymerase chain reaction (RT‐PCR) with the iScript cDNA Synthesis Kit (Bio‐Rad, CA, USA). Previously described human primers for cell differentiation were used^31^ (see Table 2). Quantitative real‐time polymerase chain reaction (qPCR) was performed in triplicate with specific primers for the genes listed in Table 2 using a CFX‐96 Real‐Time PCR system thermal cycler and SYBR green Mastermix (Bio‐Rad, CA, USA). The expression levels of glyceraldehyde‐3‐phosphate dehydrogenase (GAPDH) were used for normalization. qPCR data were analysed using LightCycler 96 Relative Quantification software (Bio‐Rad).

**TABLE 2:** Studied genes and respective primers employed for quantitative real‐time PCR (qPCR) analysis.

Gene	Host	Forward Primer 5′ → 3′	Reverse Primer 5′ → 3´	Product length (bp)
Simian virus 40 large T antigen (SV40LT)	–	GGAAAGTCCTTGGGGTCTTC	CTGACTTTGGAGGCTTCTGG	298
Collagen type II alpha 1 chain (COL2A1)	Homo sapiens	TTTCCCAGGTCAAGATGGTC	TCACCTGGTTTTCCACCTTC	498
Collagen type X alpha 1 chain (COL10A1)	Homo sapiens	CTGGACCGGCTGGAATTTCT	GCAAGCCTGGTTTCCCAAAG	471
Bone gamma‐carboxyglutamate protein (OCN)	Homo sapiens	GTGCAGAGTCCAGCAAAGGT	CTAGCCAACTCGTCACAGTC	175
Runt related transcription factor 2(RUNX2)	Homo sapiens	TATGAAAAACCAAGTAGCAAGGTTC	GTAATCTGACTCTGTCCTTGTGGAT	336
Lipoprotein lipase (LPL)	Homo sapiens	TACAGGGCGGCCACAAGTTTT	ATGGAGAGCAAAGCCCTGCTC	299
Peroxisome proliferator‐activated receptor gamma (PPARƳ2)	Homo sapiens	GACCACTCCCACTCCTTTGA	CGACATTCAATTGCCATGAG	257
Glyceraldehyde‐3‐phosphate dehydrogenase (GAPDH)	Homo sapiens	CGCGGTTCTATTTTGTTGGT	AGTCGGCATCGTTTATGGTC	231
C/EBP homologous protein (CHOP)	Rat	GAGTCTCTGCCTTTCGCCTT	AGCTGTGCCACTTTCCTCTC	389
Tumour protein P53 (P53)	Rat	CTGGACGACAGGCAGACTTT	GACAGGCACAAACACGAACC	217
B‐cell lymphoma 2 (BCL‐2)	Rat	TCGCGACTTTGCAGAGATGT	CAATCCTCCCCCAGTTCACC	116
Zonula occludens 1 (ZO‐1)	Rat	TCGGAGCTCGGGCATTATTC	CAGGGCACCATACCAACCAT	310
Binding immunoglobulin protein (BIP/GRP78)	Rat	TTCCGAGGAACACTGTGGTG	TCTCGGCGTCATTGACCATC	330
Ferritin heavy chain (FTH1)	Rat	GCCAGAACTACCACCAGGAC	AAGATTCGTCCACCTCGCTG	217
Glutathione peroxidase 4 (GPX4)	Rat	GACGCCAAAGTCCTAGGAAGC	CGGTTTTGCCTCATTGCGAG	221
GAPDH	Rat	ACGGGAAACCCATCACCATC	AGTGATGGCATGGACTGTGG	338

### 2.6 Western blot analysis

Cultured cells were washed with PBS and lysed in protein lysis buffer containing protease and phosphatase inhibitors (Sigma‐Aldrich, MO, USA). The supernatant was collected, and the total protein concentration was measured using a BCA Protein Assay kit (Thermo Scientific, MA, USA). Protein samples were separated by SDS–PAGE using 10% polyacrylamide gels and transferred onto PVDF membranes. The membranes were blocked and incubated with primary antibodies overnight, followed by incubation with horseradish peroxidase‐conjugated secondary antibodies (Table 1). Protein signals were imaged using a ChemiDocTM Imaging System (Bio‐Rad, CA, USA) and quantified with ImageJ software (NIH). Ponceau S Solution (Abcam, MA, USA) was used for visualizing total protein on PVDF membranes.

### 2.7 Statistical analysis

Data are presented as mean ± standard error of the mean (SEM) and analysed using GraphPad Prism software (Version 9). The Student's *t*‐test compared the means of the two groups, while ANOVA followed by a post hoc test compared means belonging to multiple groups. Statistical significance was defined as *p* < 0.05, and further categorized as *p* < 0.0001, *p* < 0.001 and *p* < 0.01.

## 3 RESULTS

### 3.1 iMSCs and iAAT‐MSCs express SV40LT and common mesenchymal stem cell surface markers

**FIGURE 1:**
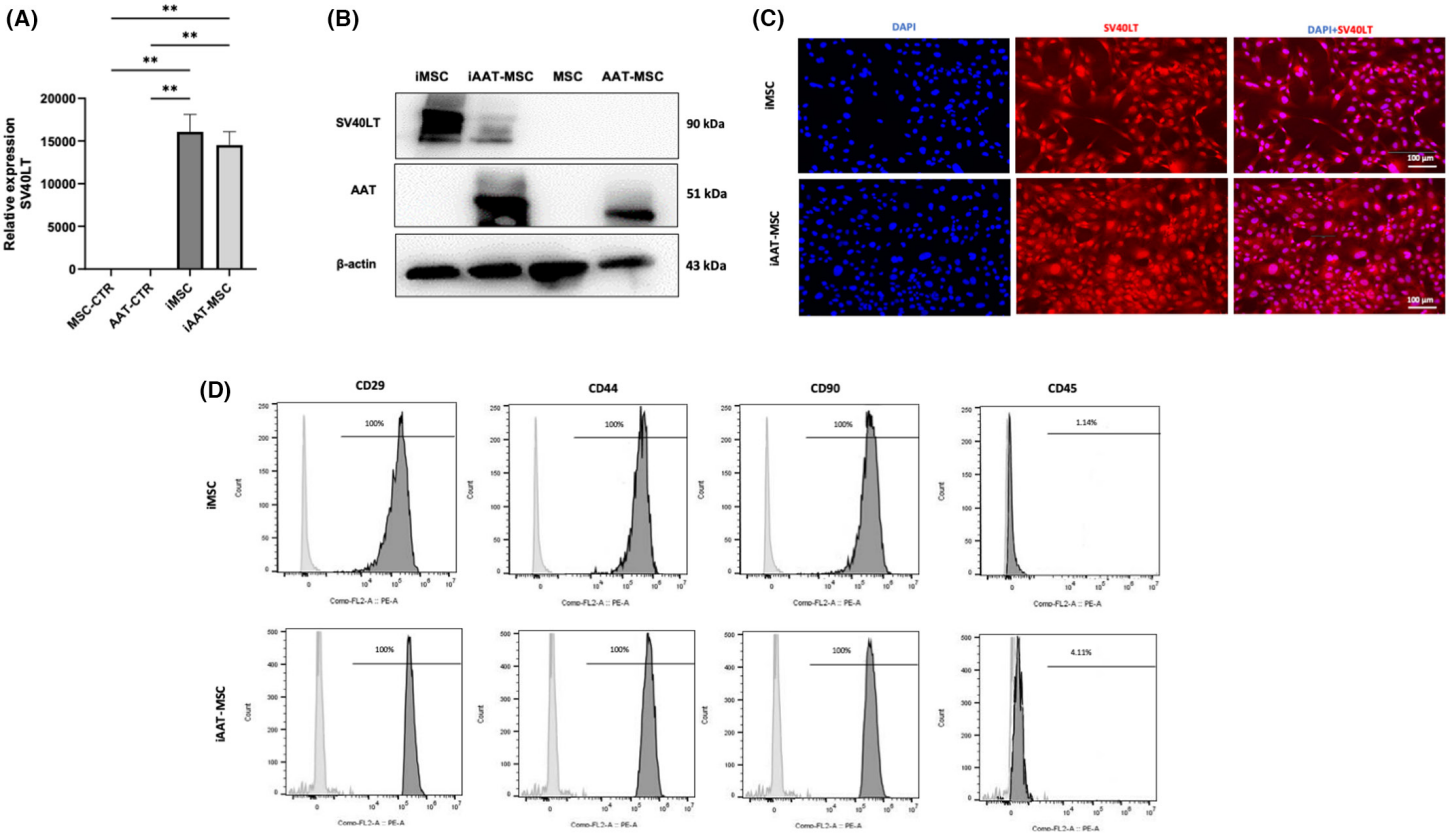
SV40LT and cell‐surface marker expression in iMSCs and iAAT‐MSCs. SV40LT and AAT expression was assessed by (A) PCR and (B) Western blot compared to primary MSC and AAT‐MSC. (C) SV40LT immunostaining of iMSC and iAAT‐MSC; SV40LT is shown in red, DAPI staining is shown in blue, scale bar = 100 μm. (D) Surface profile analysis of the cells (P8) using flow cytometry. Representative FACS analysis of iMSC‐ and iAAT‐MSC‐defining surface positive marker panel (CD29, CD44 and CD90) and CD45 as a surface negative marker. Grey histograms represent unstained controls, and the black overlays represent each antigen; percentages of positive cells are shown within histograms. Data represent mean ± SEM of *n* = 4; The *p*‐values were calculated using one‐way ANOVA; **p < 0.01. CTR, control.

We first measured the expression of SV40LT in control and immortalized cells. mRNA and protein expression of SV40LT were detected in transduced iMSCs and iAAT‐MSCs, but not in control MSCs or AAT‐MSCs, while AAT protein was detected in AAT‐MSCs and iAAT‐MSCs only (Figure 1A,B). Immunostaining further confirmed successful transduction with SV40LT, as indicated by red fluorescence specifically observed in the cell nucleoli (Figure 1C). Flow cytometric results demonstrated high expression of mesenchymal cell markers CD29, CD44 and CD90 (>95%), and low expression of haematopoietic stem cell marker CD45 (<5%) in the immortalized cell lines (Figure 1D). This data indicates the successful establishment of iMSCs and iAAT‐MSCs, which maintain the characteristic gene expression of mesenchymal stem cell markers.

### 3.2 iMSCs and iAAT‐MSCs retain MSC morphology and show a low percentage of senescent cells

**FIGURE 2:**
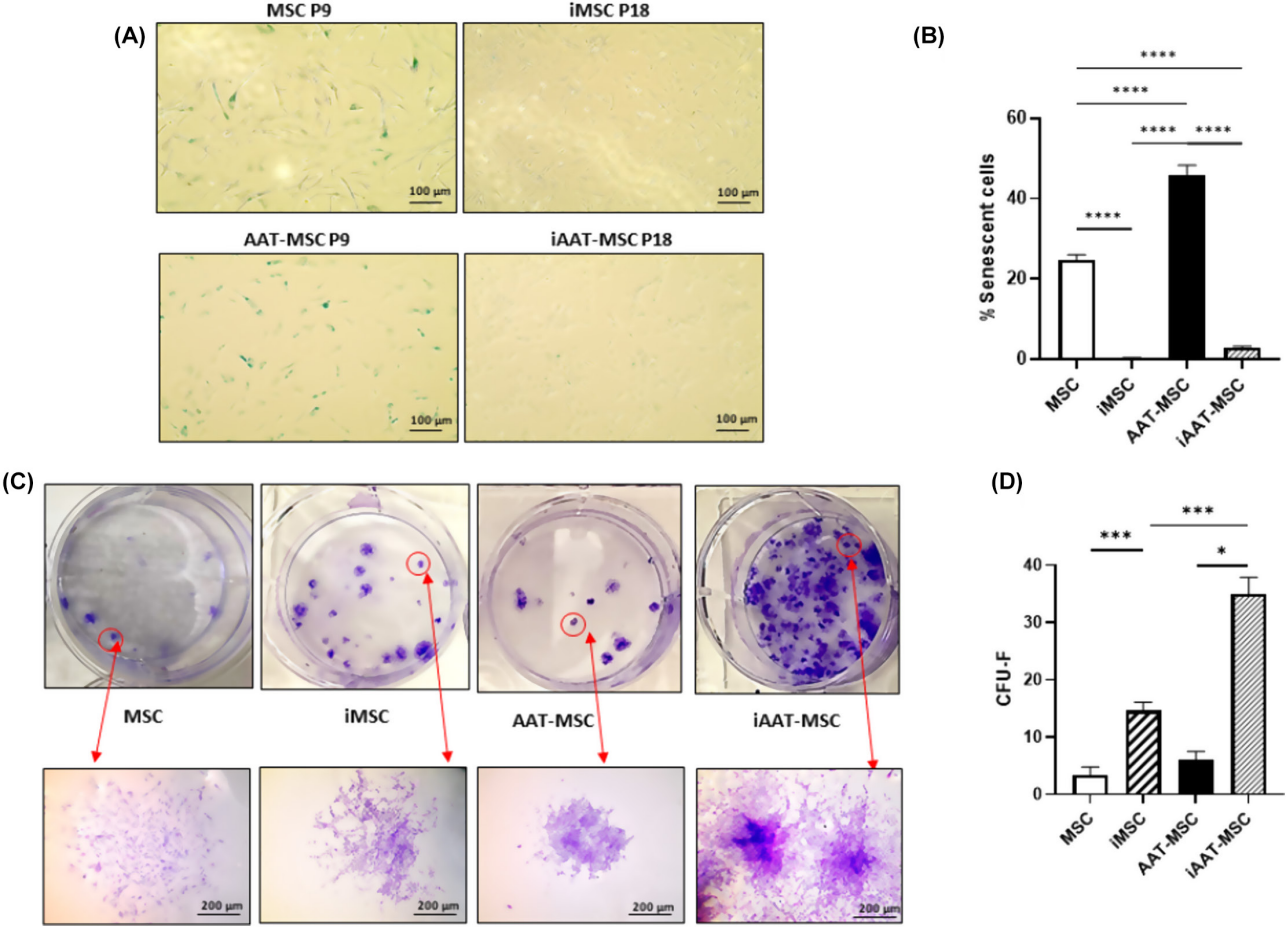
SA‐β‐gal and crystal violet staining to assess senescence and clonogenic potential of iMSCs and iAAT‐MSCs. (A) SA‐ß‐Gal stained iMSC, iAAT‐MSC and MSCs and AAT‐MSCs by phase‐contrast microscopy. SA‐ß‐Gal activity is shown in blue, scale bar =100 μm. (B) MSC morphology of iMSCs, iAAT‐MSCs, MSCs and AAT‐MSCs and percentage of senescent cells (ß‐Gal staining) at Passage 18. (C) Representative plates and total CFU‐F in iMSC, iAAT‐MSC, MSCs and AAT‐MSCs stained with crystal violet, scale bar = 200 μm. (D) Numbers of colonies at 14 days after culture in all groups of cells. The error bars represent mean ± SD for each cell line (*n* = 3); The *p*‐values were calculated using one‐way ANOVA; *p < 0.05, ***p < 0.001, ****p < 0.0001.

Late‐passage MSCs have been observed to become a heterogeneous population with a high percentage of senescent cells.^32^ iMSCs and iAAT‐MSCs showed low senescence‐associated activity even at passages 18 (Figure 2A,B). Specifically, 0.2 ± 0.5% of the cells in iMSC and 3.6 ± 1.5% in iAAT‐MSCs at P18 were SA‐β‐gal‐positive. In contrast, in primary MSCs and AAT‐MSCs at P9, 24.7% and 45.6% of cells were positive for SA‐β‐gal activity, respectively (Figure 2B), suggesting iMSCs and iAAT‐MSCs have reduced senescence.

CFU‐F assays showed iMSCs and iAAT‐MSCs had improved colony forming abilities compared to MSCs (Figure 2C). The frequency of CFU‐Fs differed significantly between iMSCs and MSCs with iMSCs forming 11.3 ± 0.3 more colonies than MSCs. Similarly, iAAT‐MSCs formed 29.0 ± 0.1 more colonies than AAT‐MSCs (Figure 2D). Furthermore, iAAT‐MSCs exhibited the most robust colony forming ability compared to iMSCs, with iAAT‐MSCs forming 20.3 ± 2.7 more colonies than iMSCs (Figure 2C,D, *p* < 0.001, ANOVA).

### 3.3 iMSCs and iAAT‐MSCs maintain capacity for multilineage differentiation

**FIGURE 3:**
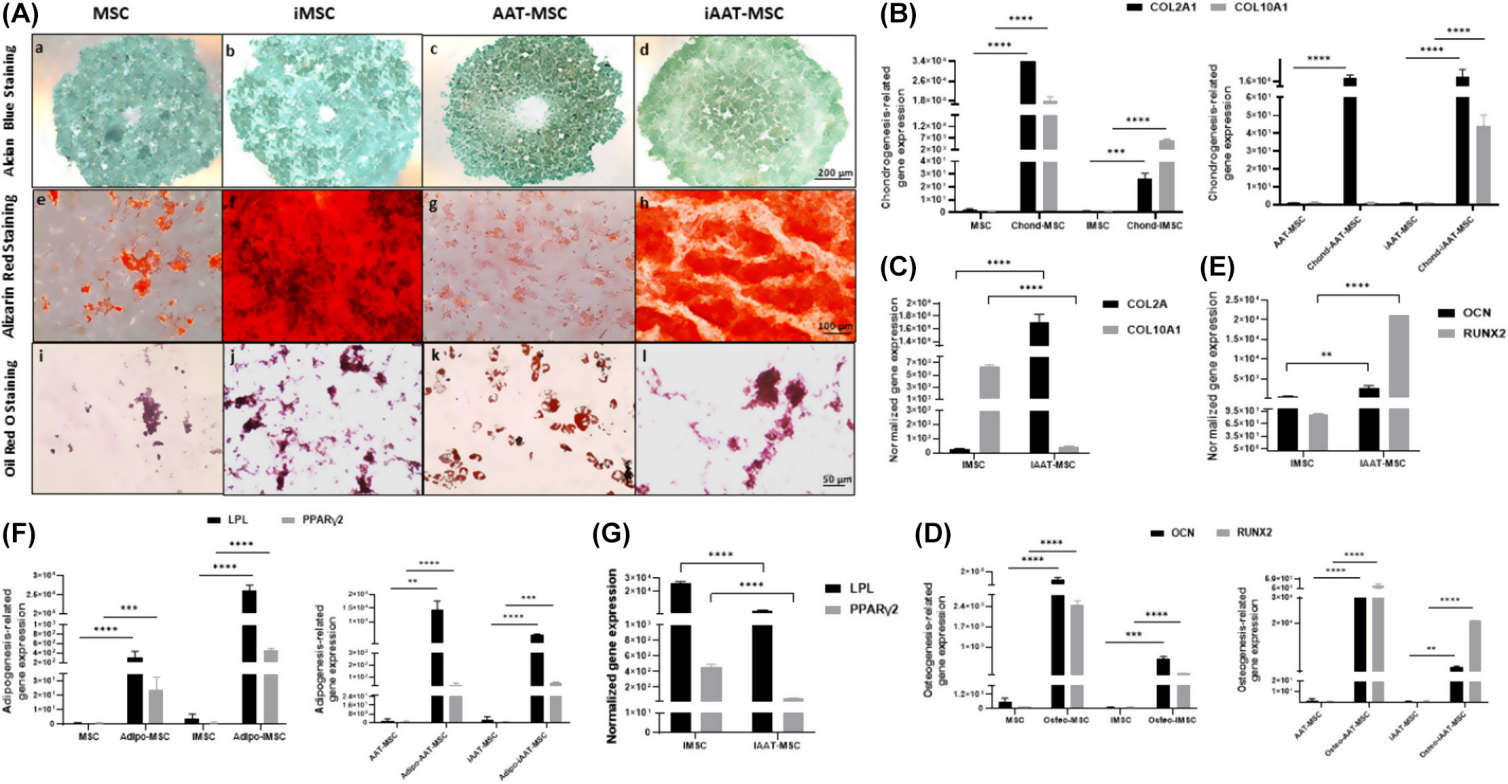
Multilineage differentiation of iMSCs and iAAT‐MSCs. (A) Chondrogenic differentiation indicated by alcian blue staining for cartilage proteoglycans immunostaining (scale bar = 200 μm); osteogenic differentiation indicated by alizarin red staining for calcium deposit and AP stain for alkaline phosphatase (scale bar = 100 μm); adipogenic differentiation indicated by oil red staining for lipoid deposits (scale bar = 50 μm). (B) Chondrogenic differentiation potential of iMSCs, and AAT‐MSCs shown by upregulation of chondrogenic markers (Col2A1 and Col10A1) assessed after 3 weeks of chondrogenesis under pellet culture. (C) Chondrogenesis‐related gene expression was shown by comparing iMSC and iAAT‐MSC. (D) Osteogenic markers (OCN and RUNX2) were upregulated after 3 weeks of osteogenesis. (E) Osteogenesis‐related gene expression was shown by comparing iMSC and iAAT‐MSC. (F) Adipogenic markers (LPL and PPARγ) were upregulated after 2 weeks of adipogenesis. (G) Adipogenesis‐related gene expression was shown by comparing iMSC and iAAT‐MSC. GAPDH was used as an internal control. Bars represent mean ± SD; The *p*‐values were calculated using Student's paired *t*‐test; **p < 0.01, ***p < 0.001, ****p < 0.0001. data represent three independent experiments.

iMSCs, iAAT‐MSCs and their non‐transduced counterparts (MSCs and AAT‐MSCs) retained multipotency (Figure 3A). Histological staining confirmed the presence of proteoglycans in chondrogenic differentiation (Figure 3a–d), calcium phosphate deposits in osteogenic differentiation (Figure 3A,e–h) and intracellular lipid droplets (Figure 3A,i–l) in adipogenic differentiation.

The expression of chondrogenic genes (COL2A1 and COL10A1), osteogenic genes (OCN and Runx2) and adipogenesis‐related genes, lipoprotein lipase (LPL) and PPARγ2 were analysed in iMSCs and their counterparts (Figure 3B–G). Chondrocyte spheroids of all cell types showed upregulated expression of COL2A1 and COL10A1 compared to undifferentiated cells (Figure 3B). iAAT‐MSCs exhibited higher expression of COL2A1 and lower expression of COL10A1 compared to iMSCs (*p* < 0.0001; Figure 3C). However, the expression of COL10A1 was higher in iAAT‐MSCs chondrocytes compared to AAT‐MSCs (*p* < 0.0001; Figure 3B). Osteoblast cultures of all cell types showed significant expression of OCN and Runx2 (Figure 3D), with higher expression in iAAT‐MSCs compared to iMSCs (*p* < 0.01 and *p* < 0.0001, respectively; Figure 3E). Adipocytes derived from iMSCs showed higher expression of LPL and PPARγ2 compared to MSCs and iAAT‐MSCs (*p* < 0.0001; Figure 3F,G). Overall, iMSCs and iAAT‐MSCs maintained multilineage potential comparable to MSCs and AAT‐MSCs.

### 3.4 iMSCs and iAAT‐MSCs inhibit early apoptosis induced by TNBS in acinar cells

**FIGURE 4:**
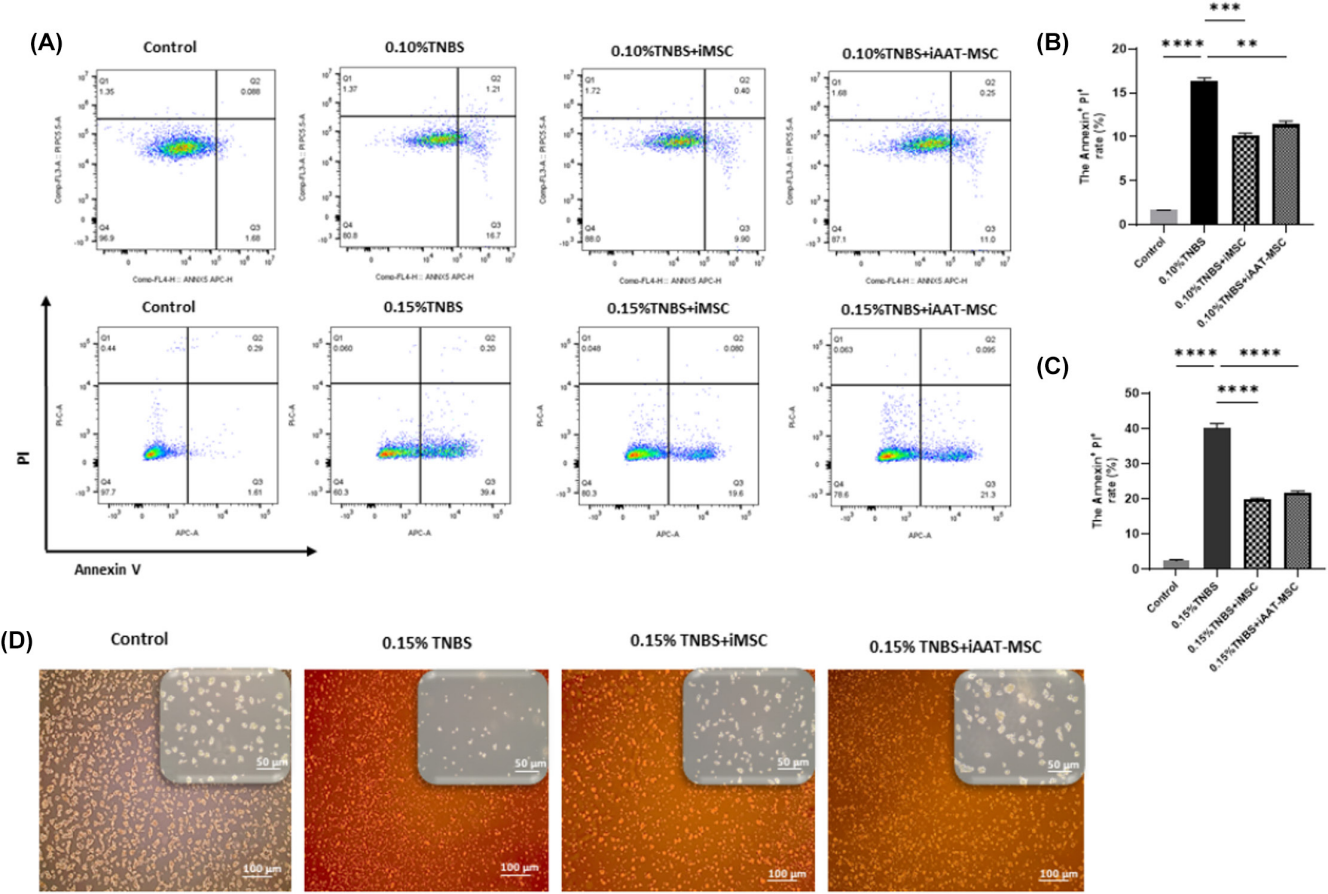
iMSC and iAAT‐MSC inhibited cell death of TNBS‐damaged acinar cells. (A) AR42J cells were cocultured with iMSCs or iAAT‐MSCs for 24 h, AR42J cells were treated with 0.10%, and 0.15% TNBS for another 24 h. AR42J cells were harvested, and then induction of apoptosis was detected using Annexin V‐FITC/PI staining assay and flow cytometry. (B) TNBS‐damaged AR42J cells (0.15% TNBS) and cocultured‐AR42J cells (with iMSCs or iAAT‐MSCs) were incubated in the conditioned medium with 5% FBS for 24 h, then observed using an inverted microscope (scale bar = 100 μm and 50 μm). (C) Quantifying the number of apoptotic cells after treatment with 0.10%, and (D) 0.15% TNBS. Data represented the mean ± SEM of three independent experiments (**p < 0.01, ***p < 0.001, ****p < 0.0001 vs. TNBS‐damaged AR42J cells).

Acinar cells were challenged with 0.1% or 0.15% TNBS. Treatment with TNBS induced a dose‐dependent early apoptosis in AR42J cells (Figure 4A). In contrast, AR42J cells cocultured with iMSCs or iAAT‐MSCs showed significantly decreased early apoptosis rates compared to TNBS treatment alone. Cell death was reduced by 6.13 ± 0.6% in the iMSC group and by 4.9 ± 0.8% in the iAAT‐MSC group compared to the 0.10% TNBS‐treated group (Figure 4A,B). The most significant effect was observed when AR42J cells were cocultured with iMSCs and iAAT‐MSCs compared to 0.15% TNBS induction alone. Cell death was reduced by 20.1 ± 1.4% in the iMSC group and by 18.3 ± 1.5% in the iAAT‐MSC group compared to the TNBS‐treated group (Figure 4A,C). In addition, AR42J cells cocultured with iMSCs or iAAT‐MSCs exhibited colony formation similar to healthy control cells, but distinct from cells treated with 0.15% TNBS that showed single‐cell morphology (Figure 4D). Based on these results, further experiments were conducted using a TNBS concentration of 0.15%.

### 3.5 iMSCs and iAAT‐MSCs alleviate ER stress and restore the reduced expression of tight junction proteins induced by TNBS

**FIGURE 5:**
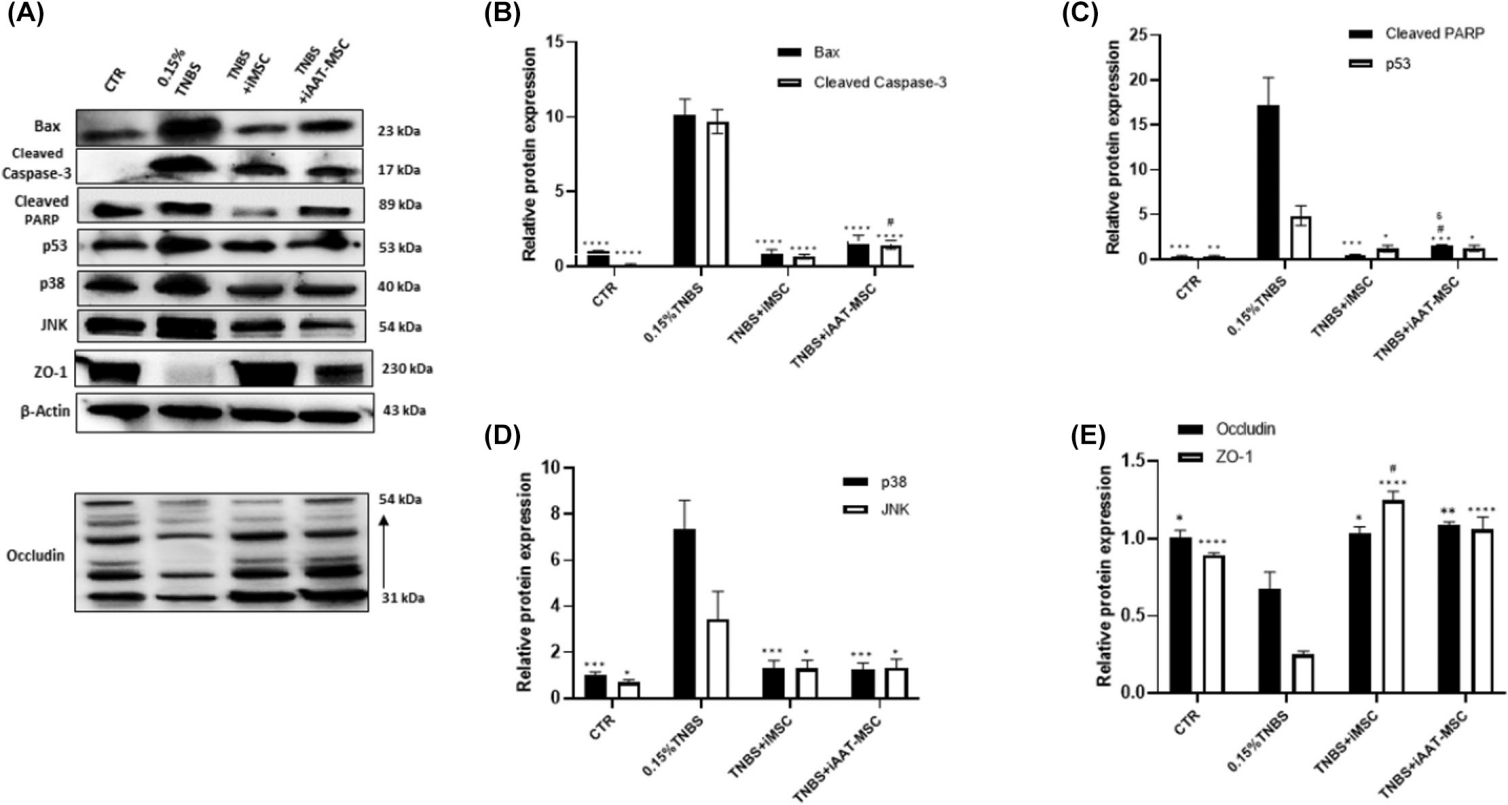
iMSC and iAAT‐MSC reduced the apoptosis markers induced by TNBS in acinar cells. (A) Represent Western blot show expression of Bax, cleaved‐caspase‐3, cleaved‐PARP, p53, p38, JNK, ZO‐1 and occludin in samples from the control, TNBS, TNBS+iMSCs and TNBS+iAAT‐MSCs groups. β‐Actin was used as the loading control. (B–D) The expression of apoptotic proteins was quantified by determining the intensities of the bands compared with those of β‐Actin. (E) Densitometric quantification of Occludin and ZO‐1 proteins in four groups of AR42J cells. For quantification of Occudin, same samples from Figure 7A were used for protein expression analysis. β‐Actin blot from Figure 7A was used for quantification. Data are presented as the mean ± SD. *p < 0.05, **p < 0.01, ***p < 0.001, ****p < 0.0001 versus TNBS group; #*p* < 0.05 versus CTR group; SP <0.05 versus TNBS+iMSC group.

**FIGURE 6:**
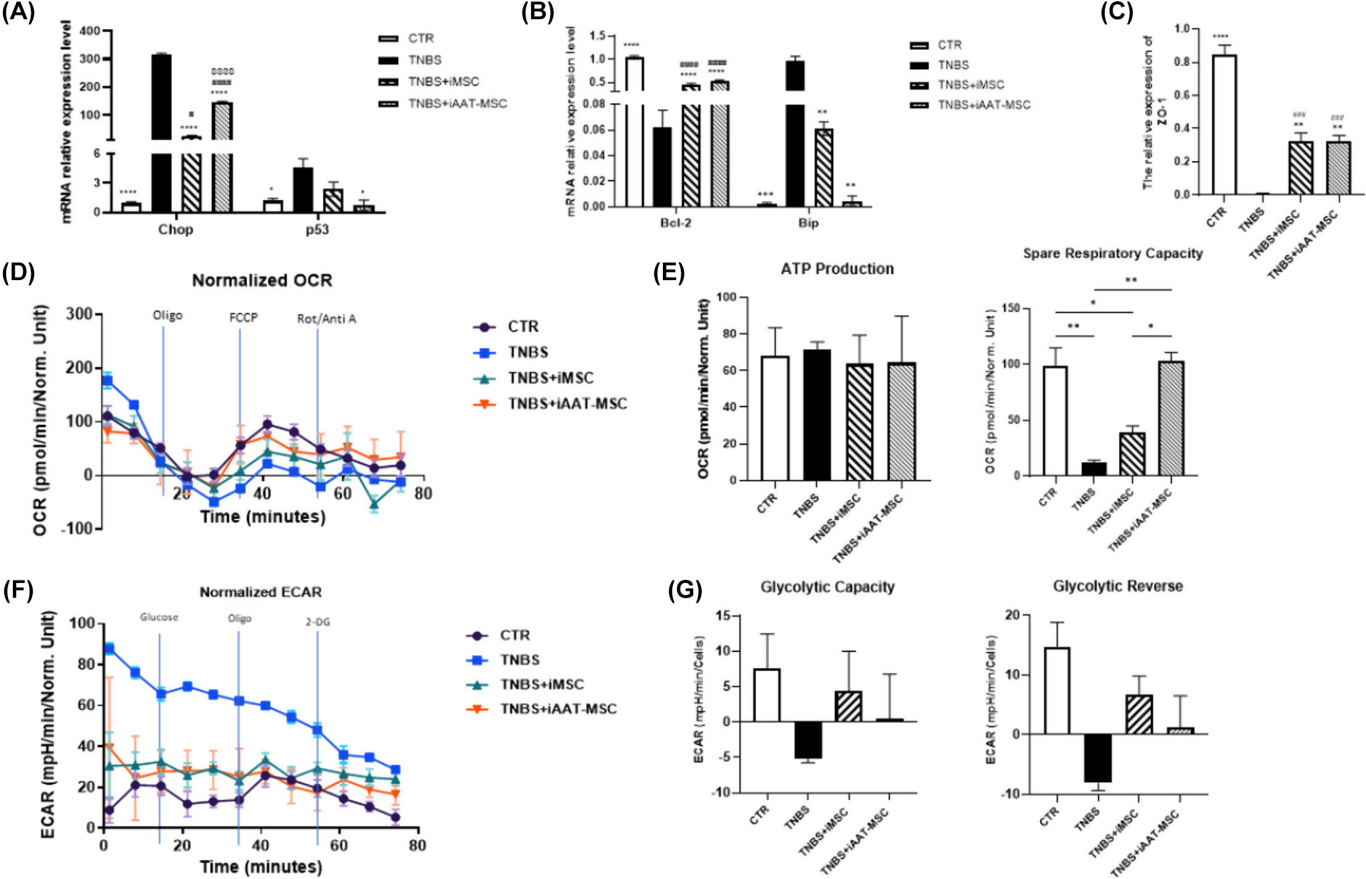
iMSC and iAAT‐MSC modulate oxidative stress and regulate mitochondrial respiration and glycolysis. (A–C) Total mRNA from TNBS treated‐AR42J cells in all groups was analysed by qRT‐PCR for the major molecules in the apoptosis signalling pathway: (A, B) Chop, p53, Bcl‐2, Bip; and (C) ZO‐1. Expression is presented as a ratio of cytokine/GAPDH. Data are presented as the mean ± SD. *p < 0.05, **p < 0.01, ***p < 0.001, ****p < 0.0001 versus TNBS group; #*p* < 0.05, ^##^ *p* < 0.01, ###*p* < 0.001, ####*p* < 0.0001 versus CTR group; SP <0.05, SSSP <0.001, SSSSP <0.0001 versus iMSC group. (D–G) After 24 h, AR42J cells were cocultured with iMSCs or iAAT‐MSCs, treated with TNBS at 0.15% concentration for another 24 h, and the dynamics of OCR and ECAR were measured. (D) Graphical representation of the OCR measurement over time; A respiratory function stress test was carried out using sequential additions of oligomycin (Oligo, 5 μM), FCCP (5 μM) and rotenone/antimycin A combined (Rot/AA, 2.5 μM) injected sequentially. (E) The effects of coculture with iMSCs and iAAT‐MSCs on the ATP‐linked OCR and spare respiratory capacity‐linked OCR were calculated from the OCR curves. Data presented as the mean ± standard deviation. *p < 0.05; **p < 0.01. (F) Graphical representation of the ECAR measurement over time; A glycolytic function stress test was carried out using sequential additions of Glucose (10 mM), oligomycin (Oligo, 5 μM) and 2‐Deoxy‐D‐glucose (2‐DG, 50 mM). (G) The effects of coculture with iMSCs and iAAT‐MSCs on the glycolytic capacity‐linked ECAR and glycolytic reserve‐linked ECAR calculated from the ECAR curves.

Several reports have suggested that treatment with TNBS induce ER stress and cell death.^33^, ^34^ We found here that the exposure to TNBS led to upregulation of pro‐apoptotic proteins including caspase‐3, Bax, PARP, p53, p38 and JNK in AR42J cells (Figure 5A–D). Coculturing with iMSCs and iAAT‐MSCs significantly suppressed the expression of Bax and cleaved caspase‐3 (*p* < 0.0001; Figure 5B). Cocultures also resulted in decreased expression of cleaved PARP, JNK, p53 and p38 induced by TNBS treatment (Figure 5C,D). In addition, TNBS treatment led to alterations in mRNA levels of CHOP, p53, BiP and Bcl‐2 in AR42J cells. Coculturing with iMSCs or iAAT‐MSCs resulted in lower expression of CHOP and BiP and increased expression of Bcl‐2 (Figure 6A,B), which might have contributed to improved acinar cell survival.

Furthermore, treatment with TNBS leads to the disappearance of tight junction proteins zonula occludens‐1 (ZO‐1) and reduced occludin expression. In contrast, iMSCs and iAAT‐MSCs significantly preserved the expression of ZO‐1 and occludin (Figures 5A,E, 6C). These data suggest that iMSCs and iAAT‐MSCs play a crucial role in reducing apoptosis at least in part by preserving tight junction integrity.

### 3.6 iMSCs and iAAT‐MSCs regulate mitochondrial respiration, ATP content in TNBS‐treated acinar cells

TNBS‐treated acinar cells exhibited mitochondrial damage, leading to a significant reduction in ATP content, indicating impaired mitochondrial function^28^ (Figure 6D,E). The spare respiratory capacity was significantly lower in TNBS‐induced AR42J cells compared to the control, being 86.2 ± 22.8 pmol/min lower than the control (Figure 6E). Furthermore, iAAT‐MSCs cocultured with acinar cells (but not iMSCs) exhibited a significantly better spare respiratory capacity compared to the TNBS‐only group, showing an increase of 102.8 ± 8.3 pmol/min over the TNBS group (Figure 6E). TNBS group also showed a high extracellular acidification rate based on ECAR analysis (Figure 6F). Additionally, treatment with iMSCs preserved the glycolytic capacity of acinar cells after TNBS treatment (Figure 6G), suggesting iMSCs and iAAT‐MSCs preserve the mitochondria function of acinar cells.

### 3.7 iMSCs and iAAT‐MSCs ameliorate ferroptosis by regulating the FTH1/PDI/GPX4 signalling pathways

**FIGURE 7:**
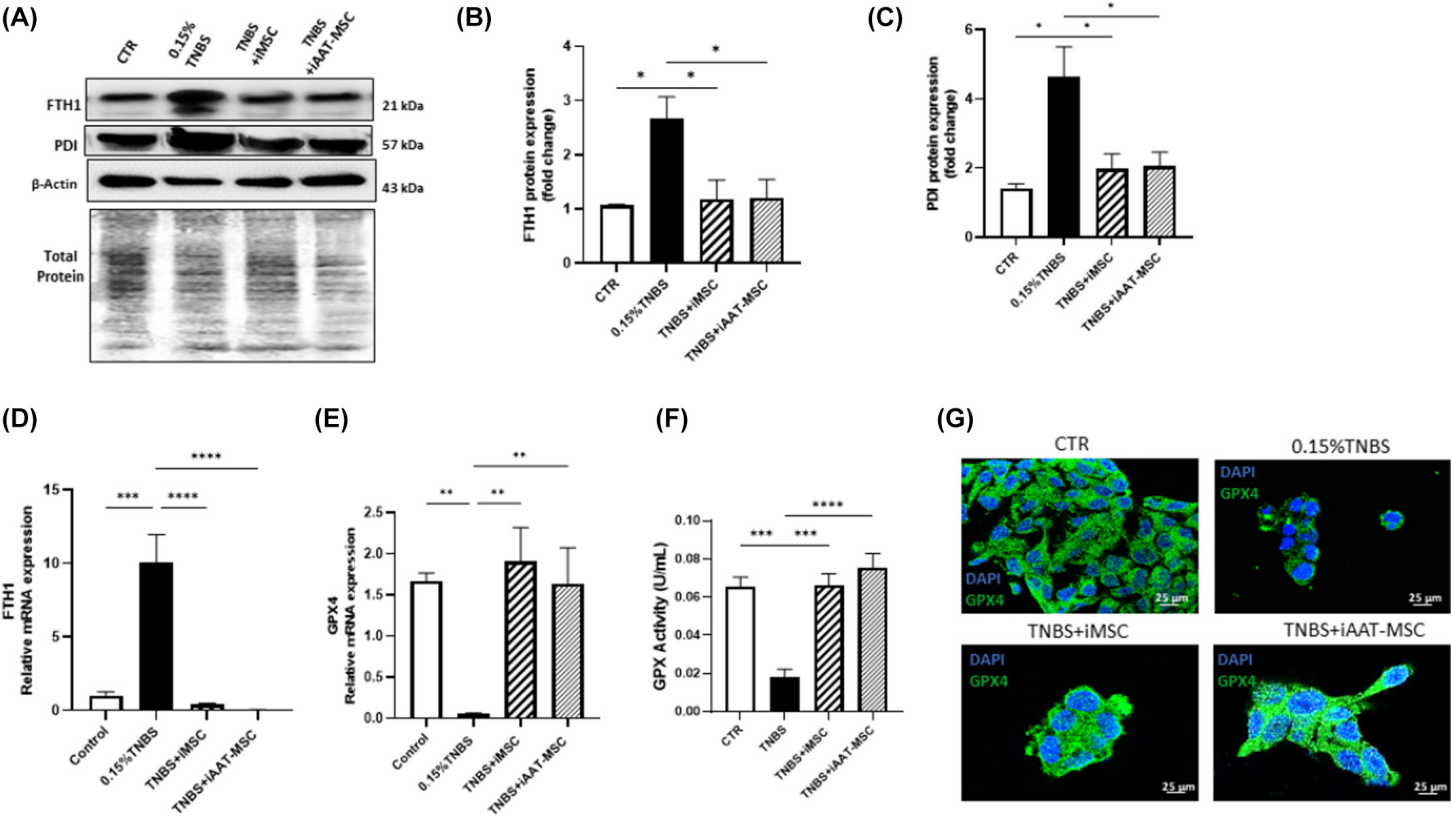
Changes in ferroptosis‐related parameters in acinar cells after 24 h of TNBS treatment. (A) Protein expression levels of FTH1 and PDI in TNBS‐induced ferroptosis in AR42J cells detected by Western blot. (B, C) Relative protein expression quantified by densitometry. (D, E). Total mRNA expression levels of FTH1 and GPX4 in three different groups of TNBS‐treated AR42J cells analysed by qRT‐PCR. GAPDH was used as an internal control to normalize the data (F) Analysis of glutathione peroxidase (GPx) activity in control (CTR) and three different groups of TNBS‐treated AR42J cells. (G) The total expression level of GPX4 in three different groups of TNBS‐treated AR42J cells was detected by immunofluorescence staining. Green fluorescence intensity reflects the protein expression level of GPX4 (scale bar = 25 μm). Data are presented as the mean ± standard deviation. *p < 0.05, **p < 0.01, ***p < 0.001 and ****p < 0.0001.

The process of ferroptosis involves primary metabolic processes such as lipid peroxidation and iron generation.^35^ We found that TNBS treatment induced ferroptosis in AR42J cells, evident by increased ferritin heavy chain (FTH1) and protein disulfide isomerase (PDI) mRNA and protein levels. However, coculturing acinar cells with iMSCs and iAAT‐MSCs effectively suppressed TNBS‐induced FTH1 and PD1 expression (Figure 7A–C), suggesting iMSCs and iAAT‐MSCs suppressed TNBS‐induced ferroptosis in acinar cells.

Next, we measured the expression of GPX4, an antioxidant defence enzyme that is functional to repair oxidative damage to lipids and a leading inhibitor for ferroptosis.^36^ GPX4 mRNA levels were increased in CP‐induced AR42J cells of TNBS plus iMSCs/iAAT‐MSCs groups compared to the TNBS group (Figure 7E). Coculturing AR42J cells with iMSCs and iAAT‐MSCs preserved GPX activity to a level like vehicle‐treated control cells (*p* = 0.0003 and *p* < 0.0001, respectively; Figure 7F). Immunofluorescence staining further confirmed the results (Figure 7G), suggesting iMSCs or iAAT‐MSCs protect acinar cells from ferroptosis via preserving GPX4 expression and activity.

### 3.8 iMSCs and iAAT‐MSCs suppress TNBS‐induced ferroptosis via modulating ROS function and iron generation in acinar cells

**FIGURE 8:**
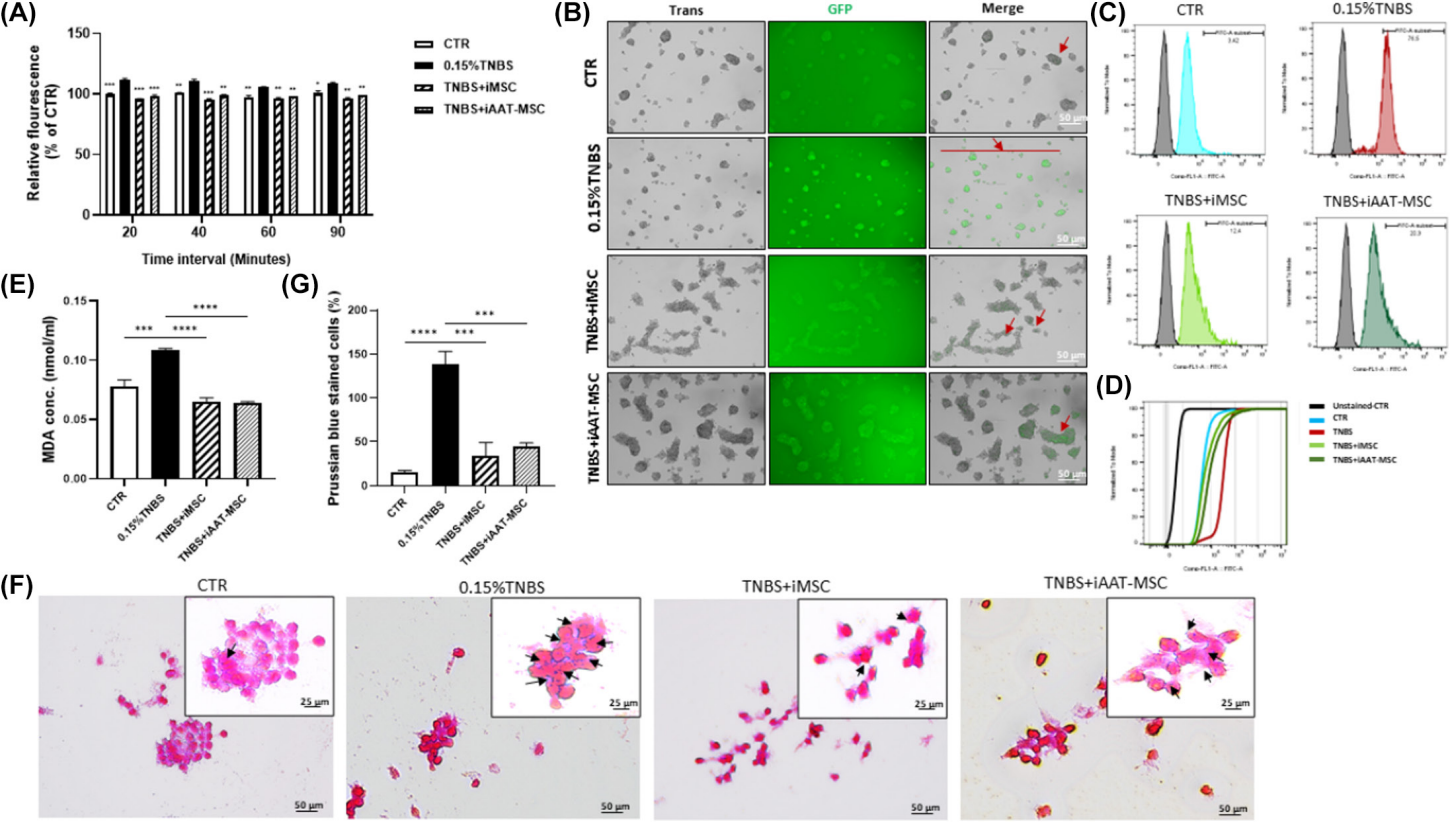
Both iMSC and iAAT‐MSC suppressed TNBS‐induced ferroptosis via modulating ROS signalling pathway in acinar cells. (A) ROS formation was measured by the relationship between the incubation time in the presence of Dihydrorhodamine (DHR)‐123 and the fluorescence at 485/528 nm after treatment. (B) Snapshots from a live cell imaging of four groups of AR42J cells treated with DHR‐123. Green fluorescence staining represents ROS production (arrows) once the probe was oxidized to rhodamine‐123 (rhod 123) (scale bar = 50 μm). (C) Flow cytometric analysis of ROS formation on live‐gated cells after treatment of AR42J cells in four groups with non‐fluorescent DHR‐123. (D) CDF curve diagram showed the comparable fluorescence signals in four treatment groups of AR42J cells with DHR‐123. (E–G) Accumulation of lipid peroxidase (MDA) and iron in four groups of AR42J cells. (E) MDA levels were determined by MDA Assay Kit. (F) Prussian blue assessment of ferrous ions accumulation in treated cells (arrows) (scale bar = 50 μm and 25 μm). (G) Quantification of iron‐positive cells from Prussian blue‐stained samples. Data represent the mean ± SEM of at least three independent experiments (*p < 0.05, **p < 0.01, ***p < 0.001, ****p < 0.0001 versus TNBS‐treated AR42J cell group).

To further confirm the capacity of preserving mitochondrial function by iMSCs and iAAT‐MSCs, we assessed ROS levels in all cell groups, as mitochondrial metabolism may affect ROS levels, and mitochondrial DNA damage could increase ROS generation.^28^, ^37^ AR42J cells treated with TNBS had significantly increased cellular ROS levels compared to control cells at different treatment times (*p* < 0.0001 after 20 min, *p* < 0.001 after 60 min and *p* < 0.05 after 90 min; Figure 8A). However, cocultures with iMSCs and iAAT‐MSCs showed significantly lower fluorescence emission compared to the TNBS group (Figure 8A). Fluorescence microscopy revealed an increased green signal in TNBS‐exposed AR42J cells, indicating enhanced conversion of DHR to Rhodamine (Figure 8B). In addition, TNBS plus iMSCs and TNBS plus iAAT‐MSCs treatments reduced the fluorescence intensity of rhodamine 123 by 12.4% and 20.3%, respectively, in comparison to 76.5% in the TNBS‐induced group alone (Figure 8C,D). These findings suggest that coculture of acinar cells with iMSCs and iAAT‐MSCs can prevent the burst of intracellular ROS caused by TNBS in acinar cells.

To confirm that ferroptosis was involved in TNBS‐induced acinar cells, we measured MDA, an end product of lipid peroxides, and iron, two indicators of ferroptosis.^14^ Treatment with TNBS induced a significant increase of MDA content when compared to controls (*p* < 0.001, Figure 8E). In contrast, the MDA content was markedly reduced in iMSCs and iAAT‐MSCs cocultured groups compared to TNBS group (*p* < 0.0001; Figure 8E). Furthermore, The iron content in the TNBS group increased significantly compared to non‐treated controls, showing a 122.8 ± 16.3% increase over control cells (Figure 8G), confirming that iron is a crucial part of intracellular lipid peroxidation in ferroptosis caused by TNBS.^35^ Coculturing with iMSCs and iAAT‐MSCs resulted in lower iron accumulation in the cell cytoplasm (Figure 8F), with significant differences in iron content between treatment groups and non‐treated cells. Specifically, the decrease in iron content was 104.1 ± 16.1% for iMSC versus TNBS‐treated cells and 93.1 ± 15.7% for iAAT‐MSCs versus TNBS‐treated cells (Figure 8G). Our findings of increased lipid peroxidation, ROS production and iron accumulation following TNBS treatment, along with their suppression by coculturing with iMSCs and iAAT‐MSCs, provide evidence supporting the involvement of ferroptosis in acinar cell dysfunction and the mechanistic insights of iMSCs and iAAT‐MSCs.

## 4 DISCUSSION

The short functional lifespan of primary MSCs significantly limits their potential for use in basic research and clinical applications, as in vitro cultures of MSCs into higher passages can lead to cell senescence. MSC immortalization is a strategy used to overcome their limited lifespan, allowing for unlimited proliferation potential.^26^, ^38^, ^39^ In this study, iMSCs at passages 18–20 exhibited higher adipogenic potential, while iAAT‐MSCs showed more significant osteogenic potential. Both cell types displayed comparable chondrogenic differentiation capacity. Similar findings have been reported in previous studies,^25^, ^27^, ^40^, ^41^ indicating that immortalization may alter the differentiation potential of MSCs but still maintain their essential characteristics. Another significant finding of this study is that treatment with TNBS induces ferroptosis in acinar cells, which may contribute to the development of pancreatitis. In addition, iMSCs and iAAT‐MSCs are suggested to protect acinar cells from TNBS‐induced ferroptosis.

Previous studies have demonstrated the beneficial effects of MSCs and AAT‐MSCs in mitigating pancreatic injury, reducing acinar cell death and inhibiting CP progression.^3^, ^5^, ^20^, ^42^ Due to the strong correlation between ER stress and apoptosis,^12^ our findings indicated a significant elevation of ER stress and cell death in the TNBS‐induced group. Treatment with iMSCs and iAAT‐MSCs effectively protected against these increases. P53, a key controller of cell apoptosis,^43^ increases the expression of Bax, a proapoptotic Bcl‐2 family member, leading to caspase‐3 activation during apoptosis.^3^, ^44^ Previous studies have consistently reported acinar cell apoptosis during CP progression.^43^, ^45^ MSCs and AAT exert cytoprotective functions via different mechanisms, although there may be overlaps in these mechanisms. For example, AAT exhibited anti‐oxidative, anti‐apoptotic and pre‐proliferative effects in human umbilical vein endothelial cells challenged by hypoxia/reoxygenation by inhibiting Rac1/PAK/p38 signalling and the p53/Bax/caspases pathways. AAT also enhances cellular integrity by upregulating ZO‐1 and occludin expression as observed in our study.^46^ Cao et al. illustrated that AAT mitigates proinflammatory cytokines—primary mediators of bone loss in oestrogen deficiency.^23^ Although MSCs are associated with antioxidant properties and can partially modulate the PI3K/Akt pathway,^47^ they exert their effects by modulating poly (ADP‐ribose) polymerase (PARP) activity by inhibiting caspases. Additionally, AAT and MSC treatments significantly reduced CHOP and BiP mRNA levels, indicating a mitigated unfolded protein response. Moreover, there are multiple studies demonstrating the anti‐apoptotic effects of AAT and MSC in different disease models.^21^ Taha et al^3^ demonstrated that BM‐MSCs effectively block free radicals induced by L‐arginine in pancreatitis treatment.

Mitochondrial dysfunction and impaired respiratory complexes were observed in acinar cells exposed to TNBS, leading to decreased cellular OCR and increased ROS levels. This finding suggests that either respiratory complexes are severely damaged or there is significant metabolic dysfunction in overall mitochondrial biochemistry.^16^, ^28^, ^48^ However, coculturing with iMSCs, especially iAAT‐MSCs, restored acinar cells' mitochondrial activity and ATP production.

Our study revealed that TNBS‐induced ER stress contributes to the development of ferroptosis, which is consistent with previous research.^49^ Ferroptosis, characterized by an imbalance between oxidation and antioxidant systems,^35^ involves various molecular factors such as iron levels, ROS, lipid ROS, GPX4 and MDA.^14^, ^16^, ^49^, ^50^ A recent study by Wei et al.^13^ demonstrated that arsenic‐induced ferroptosis via the mitochondrial ROS‐autophagy pathway contributes to pancreatic dysfunction.

In our study, superoxide free radicals initiated iron‐dependent cell death, a process modulated by the FTH1/PDI/GPX4 system (Figure S1). PDI plays a critical role in ferroptosis by contributing to the accumulation of lipid ROS.^35^ Damage to the GPX repair system leads to a lethal accumulation of ROS, and lipid peroxidation promotes ROS production.^14^, ^32^, ^51^ MSCs and iAAT‐MSCs maintain intracellular glutathione levels by directly or indirectly upregulating the expression of GPX4 to inhibit ferroptosis.^52^ Our findings further support the therapeutic effects of iMSCs and iAAT‐MSCs in inhibiting the ferroptosis pathway in the TNBS‐induced acinar cell death.

iAAT‐MSCs showed better colony‐forming ability (Figure. 2C), higher expression of chondrogenic and osteogenic genes (Figure. 3D,E), and also higher respiratory capacity after TNBS treatment, which indicates the ability of the cells to respond to an energetic demand (Figure 6E).^53^ Therefore, iAAT‐MSCs notably restored mitochondrial respiration in acinar cells more significantly than iMSCs following TNBS treatment. Since AAT is characterized as a multifunctional protein with proteinase inhibitory, cytoprotective and anti‐inflammatory properties,^23^, ^54^ we anticipated to observe a more significant reduction in apoptosis and ferroptosis in the iAAT‐MSCs group compared to iMSC in our in vitro TNBS‐treated model. However, both treatment groups showed comparable effectiveness in reducing TNBS‐induced inflammation and cell death. This could occur if the immortalization process enhanced the protective effects of iMSC, or the numbers of cells used were too high, making it difficult to see additional benefits of AAT‐MSC in our model system.

While immortalized cells may present challenges in clinical application, such as the risk of malignant transformation or significant phenotype changes due to genetic modification or extended cultivation, they offer several advantages over primary cells. These include generating more significant quantities of cells over extended periods, ease of genetic modification and consistent experimental results without donor variability.^55^ While most studies have focused on using immortalized cell lines in vitro,^24^, ^25^, ^27^, ^56^ examples demonstrate their effective use in vivo in rats and humans.^57^, ^58^ An et al. showed that immortalized rat astrocytes expressing galanin exert significant antinociceptive properties in treating pain in a rat model of sciatic nerve injury when implanted in the subarachnoid space of the lumbar spinal region.^57^ In a phase I clinical trial, the safety of intracranial administration of retrovirally transduced immortalized neural stem cells expressing cytosine deaminase was demonstrated in glioma patients.^59^ Although limited by the number of participants, the study showed overall safety, minimal side effects and low survival of these cells in a tumour area post‐chemotherapy, attributing the low oncogenic potential to the continuous presence of cytotoxic 5‐fluorouracil in the environment. Nevertheless, to overcome barriers hindering the translation of immortalized cells into clinical application, non‐viral transfection methods using chemical or physical systems to deliver interest genes into cells can be applied.^55^ These may include using lipid nanoparticles, single‐chain cyclic polymer, poly(amidoamine) dendrimers and other methods for gene delivery.^60^

Our study confirms the fundamental characteristics of bone marrow‐derived iMSCs and iAAT‐MSCs, including their localization and multilineage differentiation potential. It also demonstrates their therapeutic potential in treating the CP phenotype in a TNBS‐induced acinar cell model. By coculturing these immortalized cells with AR42J cells, we found that the activation of regulatory mechanisms of apoptotic and ferroptotic cell death in AR42J cells was inhibited. Thus, our findings provide valuable insights into the pathogenesis and treatment of CP, suggesting that iMSCs and iAAT‐MSCs may represent a promising target for CP treatment.

## AUTHOR CONTRIBUTIONS

**Sara Shoeibi:** Formal analysis (lead); investigation (lead); writing – original draft (lead). **Erica Green:** Formal analysis (equal); investigation (equal). **Hua Wei:** Formal analysis (equal); investigation (equal). **Wenyu Gou:** Formal analysis (equal); investigation (equal). **Charlie Strange:** Conceptualization (equal); writing – review and editing (equal). **Hongjun Wang:** Conceptualization (equal); funding acquisition (lead); project administration (lead); supervision (lead); writing – review and editing (equal).

## FUNDING INFORMATION

This work was supported in part by the National Institutes of Diabetes and Digestive and Kidney Diseases (R01DK105183, R01DK120394, R01DK118529, R01DK125464 and UG3DK136705) and the U.S. Department of Veterans Affairs (VA‐ORD BLR&D Merit I01BX004536).

## CONFLICT OF INTEREST STATEMENT

The authors claim there is no conflict of interest.

## Supporting information


Figure S1.

## Data Availability

Data will be available upon request from the corresponding author.
