# Supplementary material for: Immortalized mesenchymal stromal cells overexpressing alpha‐1 antitrypsin protect acinar cells from apoptotic and ferroptotic cell death
Source: J Cell Mol Med. 2024 Oct 28;28(20):e70093. doi: 10.1111/jcmm.70093 (PMC11518823; doi:10.1111/jcmm.70093)
Supplement: Supplementary file 1 — Figure S1. [file JCMM-28-e70093-s001.docx]

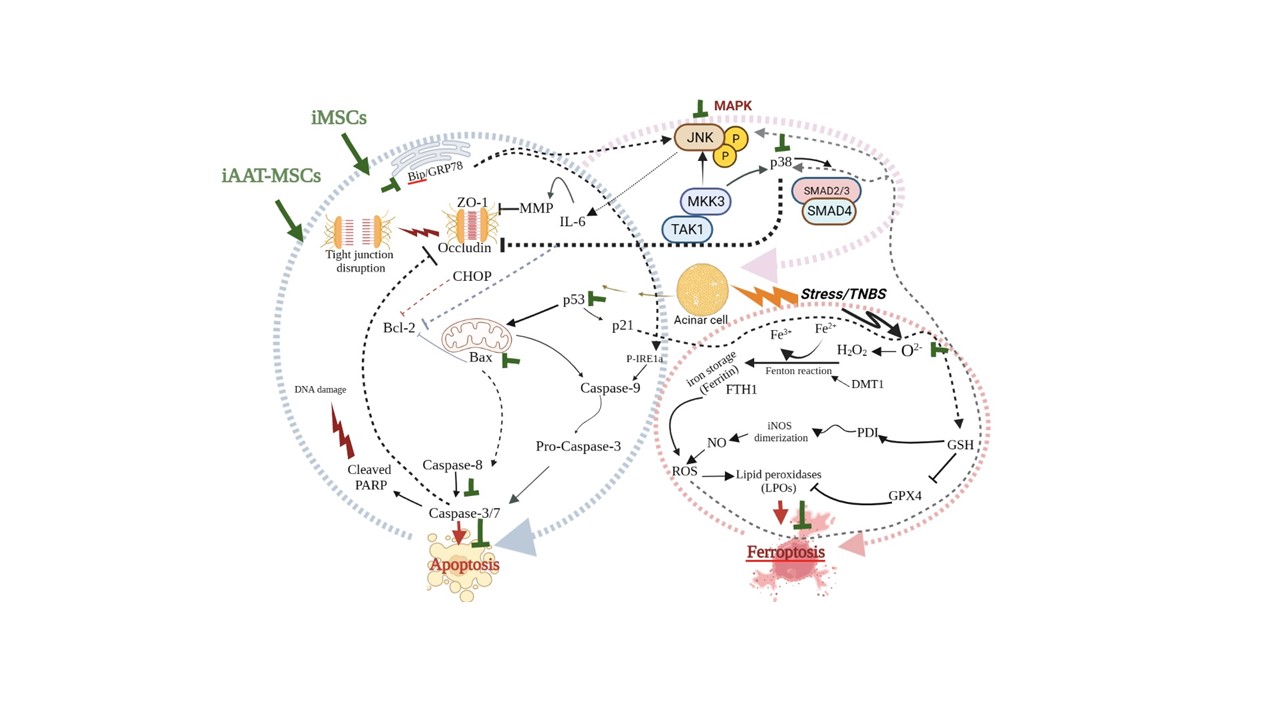


**Supplementary Figure 1. Summary of beneficial effects observed upon co-culturing immortalized-iMSCs and iAAT-MSCs with acinar cells in the current study.** TNBS-induced oxidative stress upregulates various apoptosis and ferroptosis markers in acinar cells. Treatment with iMSCs and iAAT-MSCs potentially attenuates the expression of selected apoptosis and ferroptosis markers (green arrows)
